# 2-[4-(Diethyl­amino)­benzyl­idene]malono­nitrile

**DOI:** 10.1107/S1600536811019295

**Published:** 2011-05-28

**Authors:** Yi Jing, Luo-Ting Yu

**Affiliations:** a School of Chemical Engineering, Sichuan University, Chengdu 610065, People’s Republic of China; b Chengdu institute of Organic Chemistry, Chinese Academy of Sciences, Chengdu 610041, People’s Republic of China

## Abstract

In the title compound, C_14_H_15_N_3_, the diethyl­amino N atom, benzene ring, olefinic bond and cyano groups form an extended conjugated system, making the mol­ecule nearly planar: the dihedral angle between the benzene ring and the best plane throught the cyano groups is 4.93 (10)°, while the dihedral angle between the benzene ring and the plane through the diethyl­amino N atom and the two attached ethyl C atoms is 9.51 (14)°. In the crystal, inter­molecular C—H⋯π inter­actions stabilize the packing.

## Related literature

The title compound is an inter­mediate in our research into anti­cancer agents. For general background to its chemistry, biological activity and use, see: Gazit *et al.* (1989 ▶).
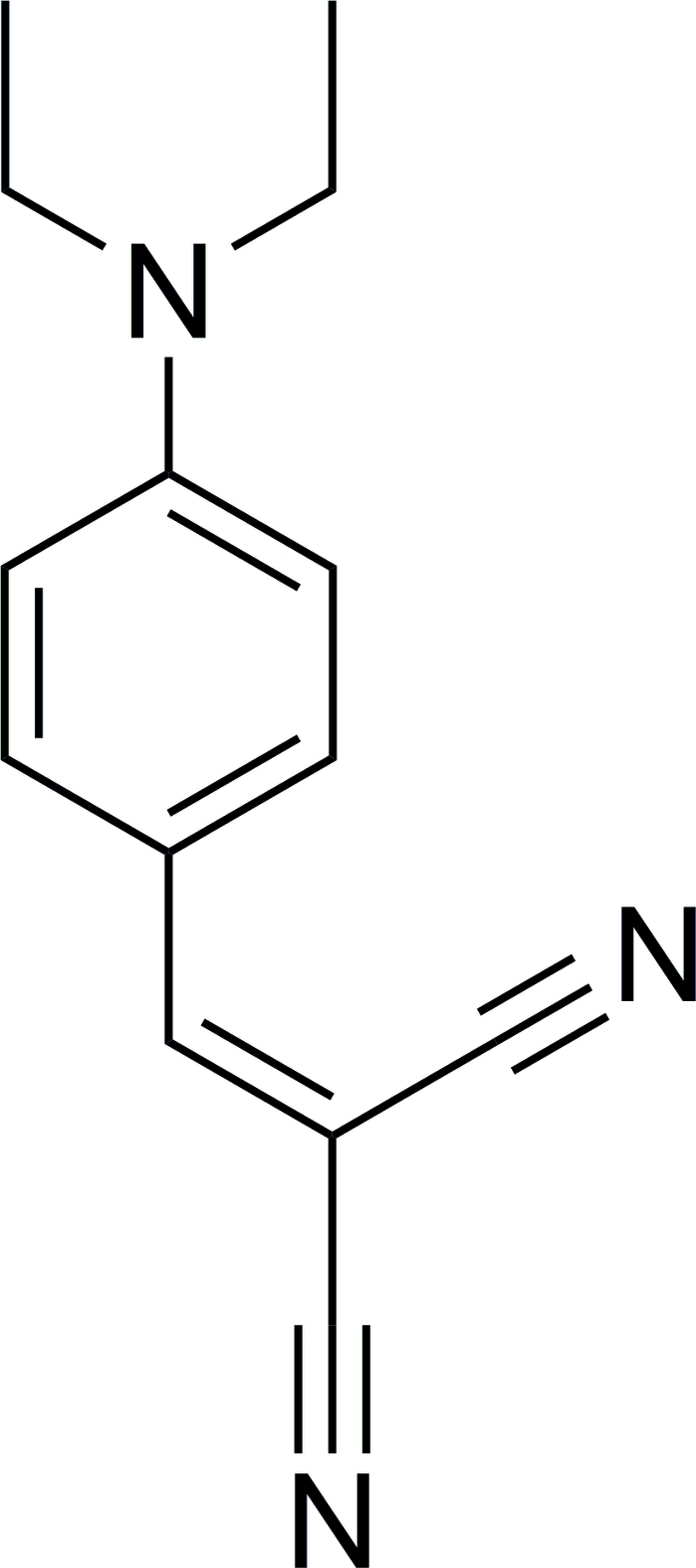


## Experimental

### 

#### Crystal data

C_14_H_15_N_3_
*M* *_r_* = 225.29
Monoclinic, 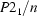

*a* = 9.2187 (2) Å
*b* = 9.4914 (2) Å
*c* = 14.5384 (4) Å
β = 97.846 (2)°
*V* = 1260.17 (6) Å^3^
*Z* = 4
Mo *K*α radiation
μ = 0.07 mm^−1^
*T* = 150 K
0.30 × 0.25 × 0.20 mm

#### Data collection

Oxford Diffraction Xcalibur Eos diffractometer
Absorption correction: multi-scan (*CrysAlis PRO*; Oxford Diffraction, 2006 ▶) *T*_min_ = 0.997, *T*_max_ = 1.000
10075 measured reflections
2577 independent reflections
2151 reflections with *I* > 2σ(*I*)
*R*_int_ = 0.022

#### Refinement

*R*[*F* ^2^ > 2σ(*F* ^2^)] = 0.038
*wR*(*F* ^2^) = 0.095
*S* = 1.03
2577 reflections
156 parameters
H-atom parameters constrained
Δρ_max_ = 0.18 e Å^−3^
Δρ_min_ = −0.18 e Å^−3^

### 

Data collection: *CrysAlis PRO* (Oxford Diffraction, 2006 ▶); cell refinement: *CrysAlis PRO*; data reduction: *CrysAlis PRO*; program(s) used to solve structure: *SHELXS97* (Sheldrick, 2008 ▶); program(s) used to refine structure: *SHELXL97* (Sheldrick, 2008 ▶); molecular graphics: *OLEX2* (Dolomanov *et al.*, 2009 ▶) and Mercury (Macrae *et al.*, 2006 ▶); software used to prepare material for publication: *OLEX2*.

## Supplementary Material

Crystal structure: contains datablocks I, global. DOI: 10.1107/S1600536811019295/vm2094sup1.cif

Structure factors: contains datablocks I. DOI: 10.1107/S1600536811019295/vm2094Isup2.hkl

Additional supplementary materials:  crystallographic information; 3D view; checkCIF report

## Figures and Tables

**Table 1:** Hydrogen-bond geometry (Å, °) *Cg*1 is the centroid of the C7–C12 ring.

*D*—H⋯*A*	*D*—H	H⋯*A*	*D*⋯*A*	*D*—H⋯*A*
C14—H14*A*⋯*Cg*1^i^	0.99	2.74	3.5154 (13)	136

Symmetry code: (i) 
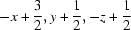
.

**Fig. 1.**
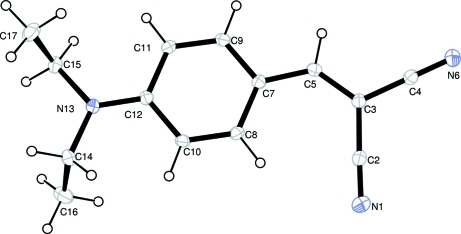
The molecular structure of the title compound, with displacement ellipsoids drawn at the 50% probability level.

**Fig. 2.**
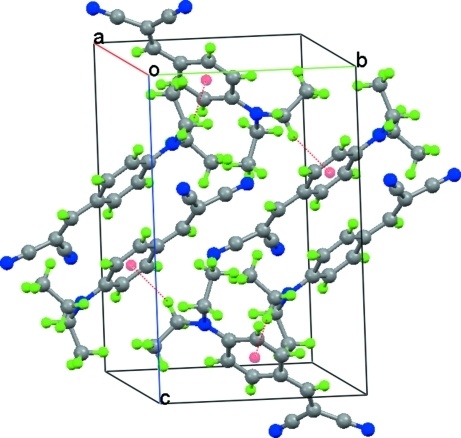
Crystal packing for the title compound, with C14—H14A···π interactions shown as dotted red lines (the centroid of ring C7-C12 is shown as a red dot).

## supplementary crystallographic information

### Comment

Cancer is a serious threat to human health. Molecular targeted therapies have created an encouraging road in the treatment of cancer in recent years. The title compound is a key intermediate in our synthetic investigations of molecular targeted anticancer agents. We report here its crystal structure.

As shown in Fig. 1, the N13 atom, benzene ring, olefinic bond and cyano-groups form an extended conjugated system, making them almost planar. The dihedral angle between the benzene plane and the best plane throught the cyano-groups is 4.93 (10)°, while the dihedral angle between the benzene plane and the plane through atoms N13, C14 and C15 being 9.51 (14)°. In the crystal, molecules are linked into a three-dimensional network by intermolecular C-H···π interactions (Fig.2, Table 1) and Van der Waals forces. Otherwise, there are no hydrogen bonds observed in the packing diagram.

### Experimental

To a solution of 4-(diethylamino)benzaldehyde (1.5 g, 8.463 mmol) and malononitrile (0.587 g, 8.886 mmol) in ethanol (25 ml) was added 4-methylmorpholine (0.9 ml). The reaction mixture was refluxed for 2 h. After cooled down to room temperature, the mixture was filtered and a red solid was abtained as the target product. Crystals suitable for X-ray analysis were obtained by slow evaporation from a solution of ethyl acetate.

### Refinement

All H atoms were positioned geometrically and refined using a riding model, with C—H = 0.95 Å (benzene C—H and C5—H5); 0.98 Å (methyl C—H) or 0.99 Å (methylene C—H) and with *U*_iso_(H) = 1.2 *U*_eq_(C) or 1.5 *U*_eq_(C) (methyl group).

### Crystal data

**Table:**

C_14_H_15_N_3_	*F*(000) = 480
*M**_r_* = 225.29	*D*_x_ = 1.187 Mg m^−^^3^
Monoclinic, *P*2_1_/*n*	Mo *K*α radiation, λ = 0.7107 Å
*a* = 9.2187 (2) Å	Cell parameters from 4336 reflections
*b* = 9.4914 (2) Å	θ = 3.1–29.2°
*c* = 14.5384 (4) Å	µ = 0.07 mm^−^^1^
β = 97.846 (2)°	*T* = 150 K
*V* = 1260.17 (6) Å^3^	Block, red
*Z* = 4	0.30 × 0.25 × 0.20 mm

### Data collection

**Table:**

Oxford Diffraction Xcalibur Eos diffractometer	2577 independent reflections
Radiation source: Enhance (Mo) X-ray Source	2151 reflections with *I* > 2σ(*I*)
graphite	*R*_int_ = 0.022
Detector resolution: 16.0874 pixels mm^-1^	θ_max_ = 26.4°, θ_min_ = 3.1°
ω scans	*h* = −11→11
Absorption correction: multi-scan (*CrysAlis PRO*; Oxford Diffraction, 2006)	*k* = −11→11
*T*_min_ = 0.997, *T*_max_ = 1.000	*l* = −15→18
10075 measured reflections	

### Refinement

**Table:**

Refinement on *F*^2^	Primary atom site location: structure-invariant direct methods
Least-squares matrix: full	Secondary atom site location: difference Fourier map
*R*[*F*^2^ > 2σ(*F*^2^)] = 0.038	Hydrogen site location: inferred from neighbouring sites
*wR*(*F*^2^) = 0.095	H-atom parameters constrained
*S* = 1.03	*w* = 1/[σ^2^(*F*_o_^2^) + (0.0409*P*)^2^ + 0.2819*P*] where *P* = (*F*_o_^2^ + 2*F*_c_^2^)/3
2577 reflections	(Δ/σ)_max_ < 0.001
156 parameters	Δρ_max_ = 0.18 e Å^−^^3^
0 restraints	Δρ_min_ = −0.18 e Å^−^^3^

### Special details

**Table:**

Experimental. CrysAlisPro, Version 1.171.34.40. Empirical absorption correction using spherical harmonics, implemented in SCALE3 ABSPACK scaling algorithm (Oxford Diffraction, 2006).
Geometry. All e.s.d.'s (except the e.s.d. in the dihedral angle between two l.s. planes) are estimated using the full covariance matrix. The cell e.s.d.'s are taken into account individually in the estimation of e.s.d.'s in distances, angles and torsion angles; correlations between e.s.d.'s in cell parameters are only used when they are defined by crystal symmetry. An approximate (isotropic) treatment of cell e.s.d.'s is used for estimating e.s.d.'s involving l.s. planes.
Refinement. Refinement of *F*^2^ against ALL reflections. The weighted *R*-factor *wR* and goodness of fit *S* are based on *F*^2^, conventional *R*-factors *R* are based on *F*, with *F* set to zero for negative *F*^2^. The threshold expression of *F*^2^ > σ(*F*^2^) is used only for calculating *R*-factors(gt) *etc*. and is not relevant to the choice of reflections for refinement. *R*-factors based on *F*^2^ are statistically about twice as large as those based on *F*, and *R*- factors based on ALL data will be even larger.

### Fractional atomic coordinates and isotropic or equivalent isotropic displacement parameters (Å^2^)

**Table:**

	*x*	*y*	*z*	*U*_iso_*/*U*_eq_	
N1	0.56613 (12)	0.33522 (13)	−0.14324 (8)	0.0415 (3)	
N6	0.83063 (12)	−0.04260 (12)	−0.12289 (8)	0.0361 (3)	
N13	0.92323 (11)	0.75238 (10)	0.22772 (7)	0.0252 (2)	
C2	0.66785 (13)	0.27997 (13)	−0.10553 (8)	0.0270 (3)	
C3	0.79456 (12)	0.20766 (12)	−0.06038 (8)	0.0235 (3)	
C4	0.81521 (13)	0.06912 (13)	−0.09534 (8)	0.0265 (3)	
C5	0.89198 (12)	0.25873 (12)	0.01132 (8)	0.0230 (3)	
H5	0.9718	0.1975	0.0308	0.028*	
C7	0.89497 (12)	0.38735 (12)	0.06192 (8)	0.0214 (3)	
C8	0.78864 (12)	0.49489 (12)	0.04813 (8)	0.0224 (3)	
H8	0.7087	0.4849	−0.0001	0.027*	
C9	1.01290 (12)	0.40925 (13)	0.13344 (8)	0.0243 (3)	
H9	1.0866	0.3390	0.1445	0.029*	
C10	0.79762 (12)	0.61324 (12)	0.10229 (8)	0.0228 (3)	
H10	0.7229	0.6825	0.0916	0.027*	
C11	1.02484 (12)	0.52810 (12)	0.18741 (8)	0.0242 (3)	
H11	1.1067	0.5391	0.2341	0.029*	
C12	0.91658 (12)	0.63493 (12)	0.17440 (8)	0.0213 (3)	
C14	0.79952 (13)	0.85020 (13)	0.22449 (8)	0.0289 (3)	
H14A	0.7991	0.8920	0.2868	0.035*	
H14B	0.7071	0.7969	0.2088	0.035*	
C15	1.05037 (13)	0.78608 (14)	0.29599 (8)	0.0297 (3)	
H15A	1.0639	0.8896	0.2984	0.036*	
H15B	1.1390	0.7440	0.2756	0.036*	
C16	0.80443 (16)	0.96798 (14)	0.15436 (10)	0.0398 (3)	
H16A	0.8031	0.9276	0.0922	0.060*	
H16B	0.8942	1.0230	0.1705	0.060*	
H16C	0.7191	1.0294	0.1551	0.060*	
C17	1.03529 (16)	0.73264 (17)	0.39255 (9)	0.0412 (4)	
H17A	1.0252	0.6298	0.3911	0.062*	
H17B	0.9484	0.7748	0.4135	0.062*	
H17C	1.1225	0.7588	0.4354	0.062*	

### Atomic displacement parameters (Å^2^)

**Table:**

	*U*^11^	*U*^22^	*U*^33^	*U*^12^	*U*^13^	*U*^23^
N1	0.0354 (6)	0.0428 (7)	0.0425 (7)	0.0053 (6)	−0.0081 (5)	−0.0064 (6)
N6	0.0351 (6)	0.0291 (6)	0.0418 (7)	0.0014 (5)	−0.0031 (5)	−0.0078 (5)
N13	0.0280 (5)	0.0248 (5)	0.0219 (5)	0.0033 (4)	0.0001 (4)	−0.0032 (4)
C2	0.0278 (6)	0.0263 (6)	0.0262 (7)	−0.0020 (5)	0.0011 (5)	−0.0047 (5)
C3	0.0252 (6)	0.0226 (6)	0.0226 (6)	−0.0006 (5)	0.0034 (5)	−0.0002 (5)
C4	0.0244 (6)	0.0276 (7)	0.0263 (6)	−0.0016 (5)	−0.0012 (5)	−0.0006 (5)
C5	0.0229 (6)	0.0214 (6)	0.0246 (6)	0.0016 (5)	0.0030 (4)	0.0032 (5)
C7	0.0226 (6)	0.0220 (6)	0.0198 (6)	−0.0008 (5)	0.0033 (4)	0.0014 (4)
C8	0.0227 (5)	0.0245 (6)	0.0192 (6)	−0.0001 (5)	−0.0002 (4)	0.0017 (5)
C9	0.0232 (6)	0.0235 (6)	0.0256 (6)	0.0041 (5)	0.0016 (5)	0.0024 (5)
C10	0.0235 (6)	0.0229 (6)	0.0216 (6)	0.0050 (5)	0.0014 (4)	0.0025 (5)
C11	0.0231 (6)	0.0270 (6)	0.0211 (6)	0.0016 (5)	−0.0017 (4)	0.0004 (5)
C12	0.0256 (6)	0.0216 (6)	0.0172 (6)	−0.0004 (5)	0.0045 (4)	0.0016 (4)
C14	0.0312 (6)	0.0294 (7)	0.0265 (7)	0.0052 (5)	0.0056 (5)	−0.0055 (5)
C15	0.0307 (6)	0.0275 (7)	0.0295 (7)	−0.0017 (5)	−0.0008 (5)	−0.0071 (5)
C16	0.0435 (8)	0.0332 (8)	0.0433 (8)	0.0120 (6)	0.0081 (6)	0.0055 (6)
C17	0.0428 (8)	0.0538 (9)	0.0246 (7)	0.0058 (7)	−0.0034 (6)	−0.0062 (6)

### Geometric parameters (Å, °)

**Table:**

N1—C2	1.1465 (16)	C10—H10	0.9500
N6—C4	1.1491 (16)	C10—C12	1.4248 (16)
N13—C12	1.3543 (15)	C11—H11	0.9500
N13—C14	1.4663 (15)	C11—C12	1.4172 (16)
N13—C15	1.4642 (15)	C14—H14A	0.9900
C2—C3	1.4343 (16)	C14—H14B	0.9900
C3—C4	1.4318 (17)	C14—C16	1.5178 (18)
C3—C5	1.3685 (16)	C15—H15A	0.9900
C5—H5	0.9500	C15—H15B	0.9900
C5—C7	1.4235 (16)	C15—C17	1.5168 (19)
C7—C8	1.4104 (16)	C16—H16A	0.9800
C7—C9	1.4134 (16)	C16—H16B	0.9800
C8—H8	0.9500	C16—H16C	0.9800
C8—C10	1.3678 (16)	C17—H17A	0.9800
C9—H9	0.9500	C17—H17B	0.9800
C9—C11	1.3700 (16)	C17—H17C	0.9800
			
C12—N13—C14	121.91 (10)	N13—C12—C11	122.45 (10)
C12—N13—C15	122.50 (10)	C11—C12—C10	116.87 (10)
C15—N13—C14	115.50 (9)	N13—C14—H14A	108.9
N1—C2—C3	178.32 (13)	N13—C14—H14B	108.9
C4—C3—C2	114.61 (10)	N13—C14—C16	113.17 (10)
C5—C3—C2	126.01 (11)	H14A—C14—H14B	107.8
C5—C3—C4	119.37 (10)	C16—C14—H14A	108.9
N6—C4—C3	179.26 (14)	C16—C14—H14B	108.9
C3—C5—H5	114.3	N13—C15—H15A	109.0
C3—C5—C7	131.43 (11)	N13—C15—H15B	109.0
C7—C5—H5	114.3	N13—C15—C17	112.85 (11)
C8—C7—C5	125.67 (10)	H15A—C15—H15B	107.8
C8—C7—C9	116.62 (10)	C17—C15—H15A	109.0
C9—C7—C5	117.71 (10)	C17—C15—H15B	109.0
C7—C8—H8	119.1	C14—C16—H16A	109.5
C10—C8—C7	121.75 (10)	C14—C16—H16B	109.5
C10—C8—H8	119.1	C14—C16—H16C	109.5
C7—C9—H9	118.8	H16A—C16—H16B	109.5
C11—C9—C7	122.42 (11)	H16A—C16—H16C	109.5
C11—C9—H9	118.8	H16B—C16—H16C	109.5
C8—C10—H10	119.3	C15—C17—H17A	109.5
C8—C10—C12	121.49 (10)	C15—C17—H17B	109.5
C12—C10—H10	119.3	C15—C17—H17C	109.5
C9—C11—H11	119.6	H17A—C17—H17B	109.5
C9—C11—C12	120.84 (10)	H17A—C17—H17C	109.5
C12—C11—H11	119.6	H17B—C17—H17C	109.5
N13—C12—C10	120.68 (10)		

### Hydrogen-bond geometry (Å, °)

**Table:**

Cg1 is the centroid of the C7–C12 ring.

**Table:**

*D*—H···*A*	*D*—H	H···*A*	*D*···*A*	*D*—H···*A*
C14—H14A···Cg1^i^	0.99	2.74	3.5154 (13)	136

Symmetry codes: (i) −*x*+3/2, *y*+1/2, −*z*+1/2.
